# Principles of Molecular Evolution: Concepts from Non-equilibrium Thermodynamics for the Multilevel Theory of Learning

**DOI:** 10.1007/s00239-024-10195-8

**Published:** 2024-08-29

**Authors:** Jens Smiatek

**Affiliations:** Institute for Computational Physics, University of Stuttgart, Allmandring 3, 70569 Stuttgart, Germany

**Keywords:** Non-equilibrium thermodynamics, Minimum entropy production principle, Evolutionary potential, Horizontal gene transfer, Mutations

## Abstract

We present a non-equilibrium thermodynamics approach to the multilevel theory of learning for the study of molecular evolution. This approach allows us to study the explicit time dependence of molecular evolutionary processes and their impact on entropy production. Interpreting the mathematical expressions, we can show that two main contributions affect entropy production of molecular evolution processes which can be identified as mutation and gene transfer effects. Accordingly, our results show that the optimal adaptation of organisms to external conditions in the context of evolutionary processes is driven by principles of minimum entropy production. Such results can also be interpreted as the basis of some previous postulates of the theory of learning. Although our macroscopic approach requires certain simplifications, it allows us to interpret molecular evolutionary processes using thermodynamic descriptions with reference to well-known biological processes.

## Introduction

The evolution of biological systems is of fundamental scientific interest. For decades, research from various disciplines has contributed to a deeper understanding of this fascinating topic. In particular, physical considerations have played a major role in recent research (Jeffery et al. 2019; Summers 2023; Kussell and Vucelja 2014). Driven by the high complexity of the problem, reductionist approaches have often been developed, attempting to describe evolutionary processes in terms of fundamental principles (Jeffery et al. 2019). In this respect, especially Schrödinger has set the direction with the introduction of the negentropy concept (Schrödinger 1951), and further approaches brought entropy as a central quantity into the focus of corresponding studies (Brooks et al. 1988; Styer 2008; Sherwin 2018; Weber et al. 1990; Sabater 2022; Martyushev and Seleznev 2006; Demetrius 2000). More specifically, the concept of negentropy or negative entropy states that the entropy of living systems decreases or remains constant, contrary to the second law of thermodynamics (Schrödinger 1951). If one loosely associates entropy with the amount of disorder, this means that living species crucially differ from inanimate systems. Such assumptions have of course led to much discussion, as both are part of a single nature which can be described by the laws of thermodynamics. As an alternative to this paradox, information theory was also often considered to explain evolutionary relations (Jeffery et al. 2019). This obvious connection was motivated by considerations about Shannon’s information entropy (Shannon 1948) and the occurrence of order during evolution (Kauffman 1993). Accordingly, also statistical approaches were used to rationalize the principles of life and order following comparable concepts (Nielsen 2006; Kauffman 1993; Ben-Jacob et al. 2000; Barato and Seifert 2015; England 2013; Perunov et al. 2016; Ramstead et al. 2018; Seifert 2012).

In contrast to such idealized approaches which are based on isolated living individuals without interactions and metabolism, network concepts for evolution are based on a more macroscopic and global description. Accordingly, a detailed description of the system behavior is replaced by simple regulating and empirical considerations, which thus drastically reduce the complexity of the assumptions. In consequence, the emphasis here is on the second law of thermodynamics and the interactions between species, which should provide an approach to the emergence of life and order in the context of pattern formation and directed processes (Jeffery et al. 2019). Pioneering work in this area was published by Eigen and Schuster 1982; Eigen 1971a, b; Gánti 2003, 1997; Prigogine et al. 1972; Prigogine and Nicolis 1971. In particular, Gánti characterized the cell as the fundamental unit of life and as a chemical automaton by means of three essential points relevant for evolution (Schwille 2015). Accordingly, the main functionalities must be a self-replicating chemical motor, e.g., the cell metabolism, a chemical information system, e.g., DNA/RNA, and a chemical boundary system such as cell membranes (Gánti 2003; Schwille 2015). These principles can thereby also be combined with descriptions of temporal evolution and the concepts of non-equilibrium thermodynamics for the consideration of dissipative systems in terms of pattern formation (Demetrius 2000; Toussaint and Schneider 1998; Nicolis and Prigogine 1971; Glansdorff and Prigogine 1971; Prigogine et al. 1972; Prigogine and Nicolis 1971).

Briefly summarized, the concepts of non-equilibrium thermodynamics are based on the study of entropy production in open systems (De Groot and Mazur 1984; Glansdorff and Prigogine 1971). This means that living species as open systems constantly interact and exchange energy or material with their environment. Moreover, it is postulated that any system subject to evolutionary processes produces entropy which is an inherent indicator of system changes. This assumption of entropy production is fundamental for non-equilibrium thermodynamics, and thus, circumvents the paradox of the negative entropy for living systems. Accordingly, it can be shown that most non-equilibrium systems relax either into equilibrium or into a stable steady state, which are characterized by a minimum of entropy production (De Groot and Mazur 1984; Glansdorff and Prigogine 1971; Lebon et al. 2008; Jaynes 1980). Hence, one no longer needs to consider negative entropies, but a minimum state of entropy production in the context of a dynamic description including thermodynamic fluxes and forces. Moreover, it can be shown that pattern formation and the occurrence of regular structures and directed processes in dissipative systems occur as a consequence of instabilities in the entropy production (De Groot and Mazur 1984; Glansdorff and Prigogine 1971; Lebon et al. 2008). One can assume that such instabilities are also relevant for the emergence of life. Accordingly, it has been shown that thermodynamic concepts can enable simplified descriptions of ordered evolutionary systems (Prigogine et al. 1972; Vanchurin et al. 2022a, b).

Recent work on evolutionary principles has also considered fitness concepts and new theories of learning (Smith 1993; Smith and Szathmary 1997; Sapp 2003; Orr 2009; Vanchurin et al. 2022a, b). A particular interesting approach was described in Refs. (Vanchurin et al. 2022a, b), where the authors introduced a thermodynamic approach in terms of the multilevel theory of learning. In a broader sense, learning was described as temporal adaption to different and multiple environmental conditions. Such learning processes can be loosely associated with genetic modification and mutation which are expressed in the phenotype of the species and, thus, enable a better adaption to changing or challenging environments. Although the similarity is not obvious, this assumption is closely related to machine learning and the corresponding improvement of predictions with respect to the minimization of loss functions that describe the differences between predicted and target values (Vanchurin 2021). In general, one of the main advantages of this theory is the introduction of thermodynamic state variables and their corresponding interpretation in the context of evolution (Vanchurin et al. 2022a, b). The authors were able to propose several laws of learning that are closely related to genetic adaptation and the corresponding evolutionary processes. Thus, molecular evolution is described as a learning process leading to optimized genetic adaptation (Vanchurin et al. 2022a). The associated evolutionary potential is closely linked to the underlying Malthusian fitness of individuals (Vanchurin et al. 2022a, b). Hence, genetic adaption interpreted as learning process tends to increase the fitness of species. Accordingly, the evolutionary species learn to adapt to environmental conditions, where progress can be described by the values of a loss function which describes the difference between the actual and the optimum state. This concept ensures a close connection between phenotype and genotype in accordance with the idea of adaptable variables. In more detail, the authors introduced different classes of adaptable and, thus, trainable variables such as the set of essential genes in a population. Thus, one can identify conserved variables that, unlike rapidly changing variables, do not have an instantaneous effect on changes of the phenotype. In contrast, there also exist certain rapidly changing and adaptable variables as essential genes which have an impact on the phenotype and, thus, lead to better environmental adaption. Despite the groundbreaking idea of this approach, it should be noted that the underlying framework is deeply rooted in standard equilibrium thermodynamics. More specifically, the authors studied evolutionary processes without explicitly considering time dependence. However, as already mentioned, the concepts of non-equilibrium thermodynamics are particularly suitable for describing these phenomena on a macroscopic level (Prigogine et al. 1972; Jeffery et al. 2019). Accordingly, a combination of the multilevel theory of learning with concepts of non-equilibrium thermodynamics to describe temporal changes would certainly be of interest in order to gain deeper insights.

In this article, we will rigorously apply a non-equilibrium thermodynamic description to the multilevel theory of learning for the study of molecular evolution processes. As main outcomes, we are able to identify the key contributions of thermodynamically described evolutionary processes and to characterize their biological meaning. The corresponding expression of entropy production for evolutionary processes depends heavily on two contributions that reveal distinct features of mutational and gene transfer effects. Furthermore, we show that species that strive for optimal adaptation to environmental conditions can be characterized by the principle of minimum entropy production. Although our approach is based on strong simplifications, empirical assumptions and a macroscopic perspective, we assume that fundamental principles of molecular evolution can be described and interpreted with sufficient accuracy.

The article is organized as follows. The next section begins with a concise introduction to the thermodynamic description of the multilevel theory of learning (Vanchurin et al. 2022a). Hereafter, we will rigorously apply various concepts of non-equilibrium thermodynamics to this approach. The obtained expressions will be discussed and interpreted in a biological context in Section 4. We conclude our considerations with a brief summary and an outlook in the last section.

## Background: Multilevel Theory of Learning

The multilevel theory of learning can be understood as a new thermodynamic approach which relies on the context of learning for the description of evolution and a connection to the origin of life (Vanchurin 2021; Vanchurin et al. 2022a, b). The respective framework introduces thermodynamic potentials and state variables but with a revised interpretation in the context of evolution (Vanchurin et al. 2022a). Moreover, certain empirical laws were introduced in close analogy to standard thermodynamics. In its most general form, the first law of learning reads

1 $$\begin{aligned} dU = TdS+ \mu dK \end{aligned},$$

where *U* denotes the average additive fitness with the evolutionary temperature *T*, the total entropy of the biological learning system *S*, the evolutionary potential $\mu$ and the number of adaptable variables *K*. In general, the previous relation is closely connected to the first law of thermodynamics which describes the internal energy change of a certain thermodynamic system. Compared to thermodynamics, the individual contributions *TdS* and $\mu dK$ have a different meaning but comparable mathematical properties. For instance, the average additive fitness *U* is also an extensive variable such as the internal energy change in thermodynamics. The entropy and the temperature have similar properties as in the first law of thermodynamics, but are brought into the context of molecular evolution. Accordingly, the entropy *S* describes the amount of order or information in the learning system while the evolutionary temperature can be loosely associated with the corresponding general impact of environmental challenges which require a specific amount of relevant information for adaption. Finally, the evolutionary potential shows similar properties as the chemical potential in thermodynamics, but is connected to the number of adaptable variables instead of the number of particles or molecules. As can be seen, the evolutionary approach does not focus on different particle species, such that one defines *K* in the context of one evolutionary and, thus, learning individual. In addition, one can note that the average loss associated with the occurrence of a single nontrainable or a single adaptable variable can be identified, respectively, with *T* and $\mu$, and the total number of nontrainable and adaptable variables with *S* and *K*, respectively. This correspondence stems from the fact that *S* and *K* are extensive and, thus, additive variables, whereas *T* and $\mu$ are intensive ones, as in conventional thermodynamics (Vanchurin et al. 2022a).

As an empirical assumption, it was postulated (Vanchurin et al. 2022a) that the number of adaptable variables reads

2 $$\begin{aligned} K = \frac{S}{b}\log N^E \end{aligned}$$

with the stochasticity factor *b* and the environmental population size $N^E$. The corresponding inverse relation

3 $$\begin{aligned} N^E \propto \exp (K) \end{aligned}$$

provides a more intuitive interpretation of Eq. (2), and we mainly use both empirical relationships for the sake of general applicability in terms of developing a generic concept of learning without the need of further specification. Thus, the previous equations imply that the effective number of variables that can be associated with genes or sites in the genome that can adapt in a given population depends on the effective population size. Accordingly, the general concept can be interpreted in two ways. The first interpretation according to Eq. (3) is that a larger number of adaptive variables can be observed in larger populations due to simple statistical considerations with regard to inheritance. Likewise, an equivalent view applies to an individual, where it is assumed that a larger set of adaptive variables is based on the size of the population (Eq. (2)). If we now interpret the amount of adaptive variables as an actual realization of an ensemble, the corresponding approach represents a probability distribution over the degrees of freedom of a single organism or a probability distribution over the entire population of organisms. In the limiting case of an infinite number of organisms, the two interpretations are indistinguishable, but in the context of actual biological evolution, the total number of organisms is only exponentially large (Vanchurin et al. 2022a). It has to be noted that the corresponding relations rely on empirical assumptions, but if we assume that *K* is also proportional to the total number of genes in the genome, the previous relation is at least qualitatively supported by comparative analysis of microbial genomes (Novichkov et al. 2009; Sela et al. 2016; Kuo and Ochman 2009; Bobay and Ochman 2017). In this context, the number of adaptable variables has been loosely associated with the set of essential genes responsible for key functions in the organism. According to this assumption, the connection between variables and their effects on the phenotype and genotype of an organism was also discussed (Vanchurin et al. 2022a).

As already mentioned, adaptable variables can be interpreted as the corresponding amount of reasonable information in the genetic material which can change over time. The consideration of adaptable variables in connection with thermodynamic state functions can be seen as one of the most significant further developments of previous interpretations. This makes it possible to place genetic evolution in the context of thermodynamic considerations and, thus, to mathematically explain spontaneous occurrence within the framework of the multilevel theory of learning. Under biological conditions, one can interpret the previous relation such that diverse and complex environments promote molecular evolution as reflected by the number of adaptable variables. In more detail, if a population of $N^E$ organisms is capable of learning the amount of information about the environment as expressed by the environmental entropy *S*, then the total number of adaptable variables *K* required for such learning scales linearly with *S* and logarithmically with $N^E$.

In more detail, Eq. (2) describes molecular evolution as the occurrence of meaningful information in the genome which can be measured as the amount of adaptable variables that change the phenotype of the individual. The introduction of the population size can be seen as the amount of already available adaptable variables. Hence, it can be assumed that large populations in particular already have a sufficiently long evolutionary history, which has already led to a certain degree of adaptation in the context of essential gene modifications. Despite the fact that one can identify different classes of adaptable variables (Vanchurin et al. 2022a, b), we here focus on variables that can change over a reasonable amount of time. Although this focus on a specific set of variables is a drastic simplification of the previous approach (Vanchurin et al. 2022a), it helps rationalize the main findings of our study for the sake of clarity. In consequence, we assume that any environmental change that drives genetic adaption requires a change in the number of adaptable variables for optimal adaption.

Furthermore, it was postulated in the previous publications (Vanchurin 2021; Vanchurin et al. 2022a, b) that the second law of learning reads

4 $$\begin{aligned} \frac{d}{dt} S \le 0 \end{aligned},$$

which means that the entropy of the learning system decreases or remains constant in equilibrium. In a simplified interpretation, this means that the amount of information either grows over time or remains constant. This clearly defines the direction of molecular evolution by means of the growth of genetic information, as this makes it clear that living organisms adapt to environmental changes over time through an increase in the number of adaptable variables.

## Non-equilibrium Thermodynamics and the Multilevel Theory of Learning: Entropy Descriptions for Molecular Evolution Processes

In general, the multilevel theory of learning is closely related to the concepts of negentropy as introduced by Schrödinger (Vanchurin et al. 2022a; Schrödinger 1951). As an extension of previous publications (Vanchurin 2021; Vanchurin et al. 2022a, b), we apply a full non-equilibrium approach to study explicit time dependencies of the underlying evolutionary processes. This explicit time dependency has not been discussed so far but is essential to understand the temporal changes in molecular evolution processes, apart from the consideration of the actual state which has already been anticipated in previous publications (Vanchurin 2021; Vanchurin et al. 2022a, b).

### Non-equilibrium Approach for the First Law of Learning

In the following, we assume that the first law of learning (Eq. (1)) is the fundamental relation for all upcoming considerations. As a reasonable starting point for any non-equilibrium description, we rewrite Eq. (1) according to

5 $$\begin{aligned} dS = \frac{1}{T}dU - \frac{\mu }{T}dK \end{aligned}$$

for all further calculations. This paraphrase can easily be justified by the fact that the change in entropy over time is fundamental to all the following conclusions in accordance with thermodynamic descriptions (De Groot and Mazur 1984; Glansdorff and Prigogine 1971; Lebon et al. 2008). Moreover, and in agreement with standard non-equilibrium thermodynamics (De Groot and Mazur 1984; Glansdorff and Prigogine 1971; Lebon et al. 2008), the properties of *S*, *U*, and *K* are evaluated in a specific isolated volume $\Omega = L^d$ with the arbitrarily chosen unit length *L* in *d* dimensions. Such a volume can be interpreted as a subsystem of a larger system with material and energy exchange. As we will later see in the context of molecular evolution, this subsystem $\Omega$ can be populated with certain interacting individuals from one biological species. Accordingly, the corresponding specific densities $\hat{s}, \hat{u}$ and $\hat{k}$ are defined by

6 $$\begin{aligned} S= & {} \int _{\Omega } \hat{s}\, d\Omega , \end{aligned}$$

7 $$\begin{aligned} U= & {} \int _{\Omega } \hat{u}\, d\Omega , \end{aligned}$$

and

8 $$\begin{aligned} K = \int _{\Omega } \hat{k}\, d\Omega . \end{aligned}$$

Insertion of these relations into Eq. (5) yields

9 $$\begin{aligned} d\hat{s} = \frac{1}{T}d\hat{u} - \sum _{i=1}^N\frac{\mu _i}{T}d\hat{k_i} \end{aligned}$$

for $i = 1,\ldots ,N$ individuals of an evolutionary species in the subsystem $\Omega$. Thus, the introduction of the subsystem with different individuals gives us the chance to consider various organisms in a well-defined local environment. For simplicity, we assume that each individual differs slightly in the number of adaptable variables. Although all individuals come from the same generation, this variation can be explained by natural mutations and the corresponding different ancestors and their inheritance rules such that the number of adaptable variables, and hence, meaningful genetic information varies. In the following, we explicitly focus on the temporal evolution of the state variables according to temporal differentiation of Eq. (9) in terms of

10 $$\begin{aligned} \frac{d\hat{s}}{dt} = \frac{1}{T}\frac{d\hat{u}}{dt} - \sum _{i=1}^N \frac{\mu _i}{T}\frac{d\hat{k}_i}{dt} \end{aligned}$$

under the assumption that the temperature and the evolutionary potential are constant. The corresponding expressions for $d\hat{s}/dt$, $d\hat{u}/dt$ and $d\hat{k}/dt$ can be derived from standard continuum equations or balance equations (De Groot and Mazur 1984). The corresponding discussion of the continuum equations in analogy to thermodynamic derivation is shown in Appendix A. Insertion of the corresponding continuum equations from Eqs. (A1) and (A2) into Eq. (10) results in

11 $$\begin{aligned} \rho \frac{d\hat{s}}{dt} = -\frac{1}{T} {\nabla }_{\textbf{L}} {\textbf{Q}} + \sum _{i=1}^N \frac{\mu _i}{T}\left( {\nabla }_{\textbf{L}} \textbf{J}_i -\breve{k}_i\right) \end{aligned}$$

with the fitness flux ${\textbf{Q}}$, the number of individuals density $\rho = N/\Omega$, the differential operator ${{\nabla }}_ L = (\partial /\partial {\textbf{L}}_j)$, the flux of adaptable variables ${\textbf{J}}$ and the source term of the adaptable variables $\breve{k}$. Vectorial quantities are marked by bold symbols. Here, it has to be mentioned that the number of individuals *N* in the volume does not necessarily has to coincide with the population size $N^E$. All vectorial quantities are denoted by bold symbols. In general, continuum equations, and thus, also Eq. (11) can be further decomposed into source and flux contributions, such that the general continuum equation for the entropy reads

12 $$\begin{aligned} \rho \frac{d \hat{s}}{dt} = - {\nabla }_{\textbf{L}} \textbf{S} + \sigma \end{aligned}$$

with the entropy flux ${\textbf{S}}$ and the internal entropy production source term $\sigma$. In more detail, the entropy production source term $\sigma$ can be interpreted as an expression for the spontaneous change of entropy in the system. A simple thermodynamic example for a physical system is internal friction that results in energy dissipation. Moreover, the entropy flux denotes all entropic changes as induced from fluxes inside and outside of the system. With a specific focus on the internal entropy production, Eq. (11) can be rearranged in terms of Eq. (12) such that

13 $$\begin{aligned} \sigma = {\textbf{Q}}\cdot {\nabla }_{\textbf{L}} \left( \frac{1}{T}\right) - \sum _{i=1}^{N} {\textbf{J}}_i \cdot {\nabla }_{\textbf{L}} \left( \frac{\mu _i}{T}\right) - \sum _{i=1}^{N}\frac{\mu _i\breve{k}_i}{T} \end{aligned},$$

which highlights that the internal entropy production within the system of volume $\Omega$ is not vanishing (De Groot and Mazur 1984). In the following, we explicitly focus on the entropy production within the system for all our upcoming discussions. Under the assumption of a constant evolutionary temperature *T*, the previous relation simplifies to

14 $$\begin{aligned} \sigma = - \sum _{i=1}^{N} \frac{{\textbf{J}}_i}{T} {\nabla }_{\textbf{L}} \mu _i- \sum _{i=1}^{N}\frac{\mu _i\breve{k}_i}{T} \end{aligned},$$

which can be regarded as the fundamental entropy production relation for molecular evolution processes in the chosen system with volume $\Omega$ for *N* individuals of a species. The assumption of constant evolutionary temperatures becomes even more reasonable under the approximation of small system sizes such that inhomogeneities can be largely ignored. Accordingly, with Eq. (14), we have established a relation which allows us to focus on entropic changes as expressed in the changes of the adaptable variable number density in combination with the gradients in the evolutionary potential density.

For further exploration of the gradients in the evolutionary potentials, we introduce a resummation due to

15 $$\begin{aligned} \sigma = - \sum _{i=1}^{N-1} \frac{{\textbf{J}}_i}{T} {\nabla }_{\textbf{L}}\left( \mu _i-\mu _{\tilde{N}} \right) - \sum _{i=1}^{N}\frac{\mu _i\breve{k}_i}{T}, \end{aligned}$$

where we introduced a reference individual $\tilde{N}$ with evolutionary potential $\mu _{\tilde{N}}$ in relation to all other individuals with evolutionary potentials $\mu _i$ for $i = 1,2,\ldots ,N-1$. Hence, the introduction of the reference individual results in vanishing total fluxes $\sum _i^{N}{} \textbf{J}_i = 0$ and $\sum _i^{N-1}{} \textbf{J}_i = -\textbf{J}_{\tilde{N}}$ which rationalizes the form of the first term in Eq. (15) (De Groot and Mazur 1984). Furthermore, it can clearly be seen that gradients between the evolutionary potential of individuals contribute significantly to the entropy production. In more detail, one can already see that the directed binary fluxes ${\textbf{J}}_i$ between the reference individual and the remaining individuals promote vanishing differences in the evolutionary potentials. As we will later see, the corresponding fluxes may lead to changes in the number of adaptable variables and, thus, the amount of genetic information in order to compensate for the differences. In principle, Eq. (15) can be regarded as a simple relation for the entropy production of molecular evolution processes in a volume $\Omega$ under the assumption of directed binary fluxes between the individuals (Eq. (15)). For a more detailed study, we include certain empirical relations for reasons of clarity.

As a first step, we focus on the source term $\breve{k}$ which accounts for the internal change in the number of adaptable variables. In a recent publication (Vanchurin et al. 2022a), it was discussed that the number of adaptable variables can be written in terms of Eq. (2). Although this is an empirical relation without any claim of full validity, we here use this expression in combination with Eq. (A2), such that

16 $$\begin{aligned} \breve{k} = \rho \frac{S}{b}\frac{d}{dt}\log N^E \end{aligned},$$

which highlights the close connection to the change of the population size $N^E$ over time. It was already mentioned that larger populations correspond to a larger number of adaptable variables (Vanchurin et al. 2022a). In simple words, this means that larger populations tend to have a larger number of adaptable variables when compared to smaller populations. This is a simple consequence of different realizations of genomes which differ in their corresponding values of the genetic information. The underlying assumption can be linked to the fact that larger populations already have a longer evolutionary history behind them and are correspondingly richer in genetic information. We adopt this idea in the following, so that the population size $N^E$ is also a good estimator of the degree of genetic adaptation of each individual within the population. Accordingly, the present state is also a reflection of the past, whereby the respective mechanisms of inheritance of the information need not be defined in more detail. In order to obtain a realistic estimator for the population size and, thus, for the degree of genetic information, we can use various empirical growth laws from the literature.

An often used empirical relation is the exponential growth law for populations of a chosen biological species (Begon et al. 2009) according to

17 $$\begin{aligned} N^E = N^E_0 e^{\omega t} \end{aligned}$$

with the growth parameter $\omega$. It has to be noted that such an approach is only valid as simplified assumption for bacterial growth and needs to be replaced by more accurate descriptions such as logarithmic growth expressions for more realistic considerations (Begon et al. 2009). However, for the sake of clarity, we discuss the further evaluation of the resulting expressions using such a simplified approach. A more realistic expression will be presented in the remainder of the article. The corresponding insertion of the exponential growth relation (Eq. (17)) into Eq. (16) and Eq. (15) yields

18 $$\begin{aligned} \breve{k} = \rho \omega \frac{S}{b} \end{aligned}$$

and

19 $$\begin{aligned} \sigma = - \sum _{i=1}^{N-1} \frac{{\textbf{J}}_i}{T} {\nabla }_{\textbf{L}}\left( \mu _i-\mu _{\tilde{N}} \right) - \sum _{i=1}^{N}\omega \rho \frac{\mu _i}{T} \frac{S}{b} \end{aligned}$$

which underlines the strong connection of the entropy production $\sigma$ with the growth rates due to the explicit occurrence of $\omega$ in the right term. In accordance, one can assume that individuals from a fast growing population or a fast growing population in the past reveal a significant amount of entropy production according to substantial increases in the amount of adaptable variables. In more detail, Eq. (19) is a rather complex expression which needs to be simplified in order to draw some general conclusions for certain population examples. Such simplifications can be attributed to vanishing growth rates and a restriction in the number of individuals in the subsystem of volume $\Omega$. The corresponding calculations are presented in the Appendix B. As can be seen for vanishing growth rates and a small number of individuals, the entropy production for these limiting cases becomes more negative or stays constant over the course of time. Accordingly, one can conclude that a large number of individuals and large growth rates are essential for a significant entropy production in terms of increasing numbers of adaptable variables and, thus, a larger amount of genetic information. As also shown in Appendix B, the full consideration of all empirical relations without any restrictions on growth rates and for *N* individuals reads

20 $$\begin{aligned} \sigma = -ab^2\, S^{n-2} \sum _{i=1}^{N-1} e^{\frac{b}{S}K_i}\frac{{\textbf{J}}_i}{T}\cdot {\textbf{u}}_{i\tilde{N}} - \frac{\omega a\, S^{n}}{T} \sum _{i=1}^{N} e^{\frac{b}{S}K_i} \end{aligned}$$

in accordance with Eq. (19). For reasons of simplicity, it is assumed that the individual factors do not change for different evolutionary species. The corresponding relation already shows the tendencies of entropy production for interacting individuals with directed non-vanishing fluxes ${\textbf{J}_i}$ and ${\textbf{u}}_{i\tilde{N}} = (\partial \Delta K_{i\tilde{N}}/\partial {\textbf{L}})$, where $\Delta K_{i\tilde{N}}$ denotes the difference in the number of adaptable variables between the reference individual $\tilde{N}$ and the remaining individuals. Moreover, as we have also discussed in the Appendix B, also single individuals in the system may influence the entropy production due to spontaneous changes in the number of adaptable variables or well-known cell division processes.

### General Expression: Entropy Production for Evolutionary Processes

In summary, we have derived a simple and general relation for the entropy production of evolutionary processes (Eq. (15)). Further manipulation of non-equilibrium expressions such as thermodynamic forces and fluxes in accordance with the derivations shown in Appendix C results in Eqs. (C14), (C15), (C16), (C17), which can be combined with Eq. (16) and Eq. (2), such that the entropy production can be written as follows:

21 $$\begin{aligned} \sigma = \sigma _{HGT} + \sigma _M \end{aligned}$$

with

22 $$\begin{aligned} \sigma _{HGT} = - \sum _{i=1}^{N-1} \frac{{\textbf{J}}_i}{T} \frac{\partial \Delta \mu _{i\tilde{N}}}{\partial \Delta K_{i\tilde{N}}}\cdot {\textbf{u}}_{i\tilde{N}} \end{aligned}$$

and

23 $$\begin{aligned} \sigma _M = - \sum _{i=1}^{N}\frac{\mu _i}{T}\frac{\rho S}{b}\frac{\partial }{\partial t}\log N^E_i \end{aligned}$$

as two separate contributions for a system with *N* evolutionary individuals. As can be seen, the entropy production is mainly governed by differences in the evolutionary potentials between the species for $\sigma _{HGT}$ and the resulting fluxes in combination with temporal internal changes in the number of adaptable variables for $\sigma _{M}$ as established from the number of ancestors or previous population sizes for reference species. For a more detailed evaluation of evolutionary entropy changes over the course of time, we focus on the entropy production rates as they will be introduced in the next subsection.

### Entropy Production Rates

As is known for standard non-equilibrium thermodynamics, entropy production rates provide estimates for the temporal evolution of non-equilibrium systems in terms of structure and pattern formation as well as vanishing orders (De Groot and Mazur 1984). Comparable conclusions can also be drawn for evolutionary systems. Thus, we aim to study the temporal evolution of biological systems in terms of changes in the number of adaptable variables and their corresponding consequences for the genetic information growth. As is further discussed in the Appendix C, one can define the entropy production density under the assumption of the exponential growth law (Eq. (17)) and $N=2$ individuals in the system $\Omega$ according to

24 $$\begin{aligned} \frac{dP}{dt}= & {} -2 \int d\Omega \left[ ab\omega \, S^{n-1} \frac{{\textbf{J}}_1}{T}\cdot {\nabla }_{\textbf{L}} (N^E_1-N^E_{\tilde{N}})\right. \nonumber \\{} & {} \left. + \frac{\omega ^2 a\, \rho S^{n}}{T} (N^E_1 + N^E_{\tilde{N}})\right] < 0 \end{aligned}$$

with the free factors *n* and *a* after consideration of Eq. (17). These factors are closely related to loss functions as was in more detail discussed in Vanchurin et al. 2022a. Accordingly, the previous relation highlights the entropy production rate for two individuals in a specific volume whose population size follows the exponential growth law. Clearly stated, it does not mean that the actual population is restricted to two species but the reference individual only interacts with one individual from the same species in the considered system. In agreement with standard non-equilibrium thermodynamics, it has to be noted that the previous relation does not fulfill the requirements of minimum entropy production (De Groot and Mazur 1984) in terms of $\lim _{t\rightarrow \infty } dP/dt < 0$ due to the properties of the exponential growth law $\lim _{t\rightarrow \infty } N^E = \infty$ (Eq. (17)). As can be seen, the minimum entropy production is only achieved for $\omega = 0$, $S=0$ or $T=\infty$ which correspond to trivial non-evolutionary conditions. In general, the principle of minimum entropy production shows that the steady state of an irreversible process, e.g., the state in which the thermodynamic variables are independent of the time, is characterized by a minimum value of the rate of entropy production (Klein and Meijer 1954; Callen 1957). Accordingly, one can assume that for any system with symmetric Onsager coefficients $L_{AB}=L_{BA}$ (more details can be found in Appendix C) which is driven out of equilibrium by applying time-independent constraints on the thermodynamic forces approaches the steady state characterized by the minimum of the entropy production functional (Eq. (21)). Such a relation is of particular interest in order to study the properties of evolutionary systems. As molecular evolution can also be interpreted as a non-equilibrium process, one can speculate that the steady state of evolution is reached in accordance with the minimum entropy production principle. According to our previous calculations, however, it comes out that populations with exponential growth do not reach the minimum entropy production state and accordingly also not a steady state. As the entropy production rate is determined by the number of adaptable variables, it can be concluded that the corresponding number also has to grow to infinity for infinite population sizes.

A more realistic expression for the change of population sizes is the logarithmic growth law (Begon et al. 2009) according to

25 $$\begin{aligned} \frac{dN^E}{dt}= \omega N^E \left( 1-\frac{N^E}{Z}\right) \end{aligned}$$

with the limiting capacity *Z*, where any growth vanishes for $\lim _{t\rightarrow \infty } N^E = Z$. Accordingly, we can calculate the entropy production rate for the first term in Eq. (C24) with $N=2$ which gives

26 $$\begin{aligned} \frac{dP}{dt} \propto -2 \int d\Omega \; a\omega b S^{n-1} \frac{{\textbf{J}}_1}{T}\cdot {\nabla }_{\textbf{L}}\left[ \Delta N^E \left( 1-\frac{\Delta N^E}{Z}\right) \right] \end{aligned}$$

in combination with Eq. (25) with the definition $\Delta N^E = N^E_1-N^E_{\tilde{N}}$. For infinite times, it can be assumed that $\lim _{t\rightarrow \infty } N^E = Z$ according to Eq. (25), such that the previous expression in terms of $\lim _{t\rightarrow \infty } \Delta N^E = 0$ vanishes. In addition, one can interpret the previous relation in such a way that there exists a restricted number of adaptable variables and, thus, genetic variation, such that convergence is reached for the population to the same amount of genetic information after an infinite time period. As is shown in the Appendix C and D, the combination of the second term from Eq. (C22) with the logarithmic growth law for one individual $N=1$ with $N^E_1 = 0$ in combination with Eq. (B6) yields

27 $$\begin{aligned} \frac{dP}{dt} \propto - 2\int d\Omega \left[ \frac{a\rho S^n}{T} \frac{\partial }{\partial t}\left( \frac{ N^E\omega (Z-1)}{e^{\omega t}+Z-1}\right) \right] \le 0. \end{aligned}$$

which results in vanishing entropy production rates for $t\rightarrow \infty$ after further evaluation. A simple generalization to $N=2$ is straightforward. Thus, it can be shown that Eq. (C22) in combination with the logarithmic growth law (Eq. (25)) results in

28 $$\begin{aligned} \lim _{t\rightarrow \infty } \frac{dP}{dt} = 0 \end{aligned}$$

which highlights vanishing entropy production rates in the limit of infinite times. In accordance, this means that the production of adaptable variables reaches a maximum value for populations with limited size, such that a stable end state in terms of minimum entropy production can be reached. As most processes tend to decreasing entropy production rates, one may speculate that also evolutionary processes reveal the same behavior. Therefore, it is important to consider a limited population size for reaching a stable steady state. Accordingly, our results have shown that logarithmic growth laws or growth laws with final convergence for population sizes lead to vanishing rates of entropy production, which corresponds to the principle of minimum entropy production for non-equilibrium thermodynamics. (De Groot and Mazur 1984). In consequence, it can be assumed that the entropy of the considered biological system reaches a steady state after a sufficient amount of time.

## Discussion

### General Remarks

The previous mathematical framework provides certain aspects for biological interpretations. Fundamental insight into evolutionary processes can be derived from considerations of the entropy production for non-equilibrium thermodynamic systems. As is known for thermodynamic processes, the study of the entropy changes over the course of time provides insights into certain aspects concerning the dynamic behavior of the system. Of particular interest is the identification of stable steady states. In accordance, we aim to identify stable steady states of molecular evolution as well as the most relevant contributions to the entropy fluxes and forces between evolutionary species. As a key observation, one can identify two contributions to the entropy production. For our upcoming discussions, Eq. (21) needs to be interpreted in terms of the actual entropy production at a given time $t_0$.

The first term $\sigma _{HGT}$ in Eq. (21) can be interpreted in terms of differences in the evolutionary potential for individuals in the subsystem $\Omega$ and the corresponding differences in the number of adaptable variables. Hence, the term ${\textbf{J}}_i\cdot {\textbf{u}}_{i\tilde{N}}$ can be interpreted as the directed net flux of adaptable variables between two evolutionary individuals to compensate for the differences..

In addition, the second term $\sigma _M$ also relies on the number of adaptable variables in terms of $\log N^E \propto K$. In contrast to the first term, this contribution can be interpreted as the individual number of adaptable variables, which does not rely on certain interactions with other evolutionary species or individuals existing at the same time. However, the actual number of adaptable variables depends on the population size or the number of ancestors or generations in the past. Such an assumption relies on the empirical assumption that populations with a large size are well adapted to environmental conditions such that the number of adaptable variables reflect their corresponding fitness as a consequence of molecular evolution in the past. As can be seen, the most important parameters for entropy production are the evolutionary potentials of interacting evolutionary individuals. Our previous discussion in the last section also highlighted that especially limiting growth laws lead to vanishing entropy production rates. Based on such results, we can conclude that finite growth is fundamental to reach a steady state of evolutionary processes with minimal entropy production.

Furthermore, our results allow for a simple interpretation of the second law of learning (Eq. (4)). Although plausible based on previous arguments, this relation has only been postulated so far. According to our considerations, we can associate this relation with the principle of minimum entropy production. Hence, it is not the entropy of the learning system that is described, but rather the convergence to optimal evolutionary states as stable steady states. In more detail, these states represent a unique characteristic of non-equilibrium phenomena, so that the original postulate of the second law of learning can be considered in a larger framework as a fundamental principle of approaching stable and steady evolutionary states.

### Biological Interpretation

In the following, we will interpret the individual contributions of the entropy production within a biological context. For our following argumentation, we ignore limited life times of species as well as complex multicellular organisms. Thus, we basically focus on single prokaryotic cells such as bacteria or archaea whose number of adaptable variables depends on the previous population size. Accordingly, we assume that the current number of adaptable variables for the considered species is to be interpreted as inheritance from the respective previous generations in terms of genetic modification.

Of particular biological interest is the entropy production expression as presented in Eq. (21). We start our interpretation with the second term $\sigma _M$ in Eqs. (21) and  (23). As was mentioned, pairwise interactions can be ignored for this term, such that any influence on the entropy production relies on the the individual contributions. Without replication or spontaneous changes in the number of adaptable variables, the entropy production remains constant, meaning that the amount of genetic information in the system does not change.

In agreement with Vanchurin et al. 2022a, we assume that the main driving factor for reaching a final stable evolutionary state is the adaption to the actual value of the environmental learning entropy *S*. Thus, for a given entropy *S*, we can assume a well-defined optimal number of adaptable variables $K_O$ that represents the optimal state of the species. If we now interpret the adaptable variables as genetic information (Vanchurin et al. 2022a), one can clearly see from Eq. (C22), that the evolutionary potential changes over time with growing *K*. In accordance with Eq. (B6) and in agreement with the discussion in Vanchurin et al. 2022a, a change of the evolutionary potential means a genetic adaption to the environment in terms of a higher Malthusian fitness. Due to the fact that such changes are solely due to the changes in the number of adaptable variables of an individual, we attribute the contributions of $\sigma _M$ without any self-replication processes to slow genetic mutation mechanisms, in line with biological considerations (Gillespie 1984; Kimura 1968; Bernstein et al. 1985; Li and Graur 1991). As was already mentioned, the actual number of adaptable variables depends on the population size of previous generations. Hence, such arguments point to the history and inheritance effects of previous predecessor prokaryotic cells with growing mutations which shared their genetic information in terms of *K* with the actual individual. If we only consider $\sigma _M$ and ignore for the moment $\sigma _{HGT}$, one can clearly see that the entropy is as long as produced as $K < K_O$. After reaching the optimal number of adaptable variables $K_O$ for a defined environmental entropy, any evolutionary driving forces such as gradients in the evolutionary potentials disappear.

The second contribution $\sigma _{HGT}$ in Eq. (21) and Eq. (22) can be interpreted as an interaction mechanism between two or more evolutionary individuals in contact with a reference individual. As can be seen, this contribution also includes fluxes ${\textbf{J}}_i\cdot {\textbf{u}}_{i\tilde{N}}$ related to the number of adaptable variables. In terms of a reasonable interpretation, we consider such pairwise interactions in combination with fluxes in the number of adaptable variables as information flows which vanish for identical evolutionary potentials. In the limit of vanishing $\sigma _{HGT}$ and for long evolutionary times, we can assume that the optimal number of adaptable variables for each individual is already reached. In combination with the mutation contributions from $\sigma _M$, the flux in the number of adaptable variables between the individuals leads to faster adaptation in combination with faster attainment of stable evolutionary states. Thus, horizontal gene transfer between bacteria, which enables rapid genetic adaptation, is a possible biological mechanism for interpreting the contributions to $\sigma _{HGT}$ (Ochman et al. 2000; Keeling and Palmer 2008). The characteristics of this gene transfer show exactly the same properties as derived for $\sigma _{HGT}$. Accordingly, it should be noted that the balance of evolutionary potentials for $\sigma _{HGT}$ is a process between randomly interacting individuals. In fact, horizontal gene transfer relies on the exchange of genetic material between two species in order to increase the overall evolutionary fitness. This indeed can be seen as a directed information flow. Hence, such a process circumvents the time-consuming molecular mutation processes due to ensemble effects of previous population achievements.

In general, the mutation rate in unicellular prokaryotes is orders of magnitude lower compared to different virus species (Drake et al. 1998). One could speculate that this accounts for nonexistent information flow such as horizontal gene transfer between virus particles. The number of adaptable variables for viruses is rather small, so it can be assumed that $\sigma _M$ is the only contribution which is efficient enough to reach the stable steady state. Such findings could also explain the relatively high mutation rates for virus particles (Drake et al. 1998). In contrast, bacteriae have a larger number of *K*, so it makes sense to interpret information flows and the corresponding interactions as a more efficient route to a stable state of equilibrium.

Furthermore, it is also worth to mention that our findings imply vanishing entropy production rates at evolutionary equilibrium (Ochman et al. 2000; Keeling and Palmer 2008). Hence, molecular evolution within a given environment can be described as nearly analogous to standard thermodynamic adaption processes. Whenever an optimal adaption is reached, the entropy production vanishes in terms of minimum entropy production principles which characterize a stable state. However, for fast changes in the environment as expressed by rapid changes in *S*, the number of adaptable variables needs to change. Hence, evolutionary processes can be regarded as comparable to thermodynamic non-equilibrium processes.

As a result, our findings indicate that molecular evolution contributes to entropy production. We can identify internal adaptation processes (mutation) and directed information flow between different evolutionary individuals (horizontal gene transfer). As long as entropy production exists, it can be concluded that the evolutionary optimal state and the corresponding number of $K_O$ have not yet been reached. In consequence, these implications show that the main drivers of evolutionary processes are gradients in evolutionary potentials and internal mutational effects. Since nature tries to reduce entropy production in non-equilibrium processes, we consider molecular evolution as the directed change of biological systems to reach stationary states with vanishing entropy production. Such findings are closely related to recent theories about dynamic kinetic stability (Pross and Pascal 2017; Pross 2011, 2005).

### Embedding in the Context of Evolutionary Theories

The description of evolutionary processes using thermodynamic and information-theoretical concepts has a long tradition (Agosta and Brooks 2020; Brooks 1994; Brooks et al. 1988, 1989). In particular, the importance of transformative processes for the maintenance of metabolism and the exchange of materials and energy has often been taken into consideration in previous phenotype-oriented approaches (Agosta and Brooks 2020). In addition, information-theoretical concepts were also developed, which focused on the temporal behavior of entropy in the context of inheritance and the consequences for evolutionary micro- and macrostates of populations (Brooks et al. 1988, 1989). For these concepts, a close relationship between entropy in evolution and statistical mechanics was also developed (Brooks et al. 1989; Agosta and Brooks 2020). In more detail, it was shown that for a system that evolves with time, thus, becoming more complex, the corresponding phase space that is needed to describe all micro- and macrostates will grow over time (Smith 1988). For an evolving population and for a given level in a physical or biological information hierarchy, the difference between the entropy maximum and the actual entropy measures the organization of the system at that given point in time (Brooks et al. 1989; Agosta and Brooks 2020). The observed informational entropy which corresponds to the expressed information content is calculated based on the observed distribution of components. In contrast, the maximum possible informational entropy represents the potential information capacity in the system in its totally relaxed state without environment constraints, where it is assumed that all components of the system being distributed equiprobably throughout the system. The mathematical framework relies on partitioned Lebesgue spaces with automorphism which reveals that entropy in this information hierarchical models show increasing and concave properties when associated with increasing organization (Smith 1988). It was discussed that both entropies converge for long times which is equivalent to a minimum entropy production as was introduced in this work (Brooks et al. 1989; Agosta and Brooks 2020). Accordingly, we can associate the corresponding entropy production in our previous considerations as the entropy production of the actual entropy of the system for evolutionary macrostates, meaning the information content of larger populations instead of individual species.

Within the context of standard evolutionary theories, three major pillars can be identified, which are referred to as Darwinism, Neo-Darwinism and hardened or modern synthesis (Agosta and Brooks 2020). Darwinism clearly corresponds to the original theory as outlined in the seminal book ’On the origin of species’ by Charles Darwin (Darwin 1964). Neo-Darwinism already was introduced very early in the twentieth century by Kellogg (Kellogg 1908) among others (Fisher 1930; Mayr 1942; Dobzhansky 1937; Huxley 1942). Modern synthesis was first proposed by Gould (Gould and Eldredge 1983; Gould 1983), who introduced the term ’hardening of the modern synthesis’ for what he perceived a progressive commitment of species to pan-adaptationism and, thus, global adaption of organisms to environment conditions as proposed by Dobzhansky (1937). From a high level perspective, the three approaches can be seen as dependent developments in a historical context and can be described shortly as follows. For more details and for a detailed discussion of the historic developments, we refer the reader to the textbook by Agosta and Brooks (Agosta and Brooks 2020).

The central idea of the Darwinian theory is that natural selection drives evolution. Organisms with advantageous traits survive and reproduce, passing those traits to their offspring. Accordingly, evolution occurs gradually through small, cumulative changes. As the molecular basis of inheritance was not known at that time, there is no link to the influence of genes and molecular mechanisms. In contrast, Neo-Darwinism combines Darwinian natural selection with modern genetics where genetic variation as the source of heritable traits is recognized. Despite its success, Neo-Darwinism faces criticism due to limitations in explaining complex traits and rapid evolutionary changes. The hardened synthesis also known as modern synthesis finally merged genetics, natural selection, and population biology and it emphasizes species adaption to specific environments. In more detail, modern synthesis also addresses gene flow between populations and it explains how traits adapt to ecological conditions such that natural selection is sharpened as a creative force. In summary, while Darwinism laid the foundation, Neo-Darwinism refined it with genetics, and the modern synthesis integrated diverse new aspects of evolution in terms of molecular biology and population dynamics (Agosta and Brooks 2020).

Moreover, it was recognized that Neo-Darwinism is primarily a theory of stasis, while Darwinism is a theory of evolution (Agosta and Brooks 2020). As mentioned as few examples (Agosta and Brooks 2020), Darwinism in terms of evolution is the interplay of the nature of the organism and the nature of the conditions but the nature of the organism is being far more important for evolutionary changes over time. For Neo-Darwinism it can be stated that evolution is adaptation by random variation to changing environments but static and equilibrium considerations are key components. A prime example for equilibrium considerations is the Hardy-Weinberg law (Hardy 1908; Weinberg 1908; Stern 1943), which states that allele and genotype frequencies in a population will remain constant from generation to generation in the absence of other evolutionary influences. Accordingly, modern discoveries such as gene shift, gene drift or mutation are ignored and general equilibrium between two states is assumed.

Further modern concepts also address questions of best adaption of species to environmental conditions. With regard to these considerations, novel approaches such as the sloppy fitness landscapes (Agosta and Klemens 2008; Agosta et al. 2010; Agosta and Brooks 2020; Brooks and Agosta 2012) mainly focus on questions concerning ecological fitting of species to certain environments and constraints. The ability to adapt ecologically provides heritable systems with crucial degrees of freedom to cope with a changing environment by exploring new options in underutilized, less preferred or previously inaccessible parts of the fitness space. The ability to move from densely populated, deteriorating or disappearing parts of the fitness space to new regions of the fitness space is the key to unlimited persistence even if this leads to reduced fitness of species. Accordingly, organisms will do what they can, where they can, when they can, within the constraints of evolutionary history as represented by inheritance and ecological opportunity (Agosta and Brooks 2020). As was assumed in Darwinism, evolutionary dynamics are the result of simple inheritance with organisms in terms of non-zero fitness wandering through a sloppy fitness space. For Neo-Darwinism, the fitness space is highly optimized with fuzzy boundaries, and organisms do not change fitness space without eliminating a less fit occupant (Agosta and Brooks 2020). However, although there are many more subtle differences between Darwinism, Neo-Darwinism and modern synthesis, certain aspects of our approach can be aligned with these standard concepts.

In general, an important concept of Neo-Darwinism is learning and adaption of highly adapted species in a narrowly optimized fitness space. Evolutionary changes due to external conditions mainly evolve as new adaptions. When conditions change, the only way to escape this narrow fitness state is to evolve new adaptations of the right kind at the right time. In contrast, Darwinism focuses on potentially inherited information accumulating faster than realized information and interacting with the environment that comprises a sloppy rather than a tightly optimized fitness space. Darwinism is about survival of the adapted, not survival of the fittest - it is about coping with change by changing, and for Darwinism the answer lies in the history of the biological context (Agosta and Klemens 2008; Brooks and Agosta 2012; Agosta and Brooks 2020).

According to the simplified considerations in our theory, we can attribute our approach as Darwinian rather than Neo-Darwinism. Although adaptive variables are mentioned, they are treated in general terms, and the properties of genes are not explicitly mentioned or considered, nor are they explicitly included in the theory. Accordingly, modern concepts of gene drifts, shifts and advanced mutations are not included in the corresponding equations, which are, thus, formulated quite generally and broadly. In addition, concepts from population genetics and population dynamics are only marginally considered. In detail, the corresponding approach concentrates on the temporal development of adaptive variables and the corresponding central driving mechanisms. Based on microstates, a macrostate entropy is defined, whereby its temporal changes are characterized by mutations or gene transfer in the context of minimum entropy production and non-equilibrium thermodynamics. Accordingly, broad adaptations to changes in external conditions are more likely to be assumed in our approach, in close agreement with the concept of sloppy fitness landscapes and corresponding broad adaptations in terms of adaptable variables. Despite this possible connection to previous evolutionary considerations, however, our theory is clearly based on information-theoretical concepts as discussed in earlier works (Brooks et al. 1988, 1989). According to our concept, molecular evolution occurs through multiple processes, and optimal adaptation is mainly determined by external constraints and environmental conditions. In contrast to earlier approaches (Brooks et al. 1988, 1989), which characterize entropy as a measure of the complexity and degree of organization of the population, entropy takes on a different relevance in our theory.

Accordingly, we do not examine the effects and characteristics of evolution, nor do we discuss various molecular biological approaches or selection mechanisms. Rather, we show that evolution must inevitably take place within the framework of an information-theoretical concept. The fundamental driving force of evolution, whose current state can be described by actual entropy production, is the long-term achievement of a minimum entropy production state which is biologically characterized by an adaptation of the population to the given environmental conditions as expressed by the close linkage between pheno- and genotype. Accordingly, evolutionary processes are treated from an information-theoretical perspective, whereby answers to questions about biological effects and actions cannot be derived from this, in contrast to previous evolutionary theories such as Darwinism, Neo-Darwinism or modern synthesis.

## Summary and Conclusion

We have presented a non-equilibrium thermodynamics approach for the study of molecular evolutionary processes. Based on earlier expressions from the multilevel theory of learning (Vanchurin et al. 2022a, b) we have introduced standard approaches from non-equilibrium thermodynamics for the calculation of the entropy production. As we have discussed, the process of evolution cannot be associated with stable equilibrium and, thus, optimum genetic adaption states, such that our approach addresses the temporal changes in the number of adaptable variables which affects the entropy production of the species. In general, we have defined entropy production and the entropy production rate for evolutionary processes using standard expressions from non-equilibrium thermodynamics in terms of generalized forces and fluxes. As a crucial approximation, we consider the number of adaptable variables as the genetic information that needs to be optimized for reasonable environmental adaption. Our results show that only two processes contribute to entropy production. We have linked the corresponding expressions to internal mutation processes and targeted information exchange in the sense of horizontal gene transfer between simple cells. Further results showed that only limited population sizes, as expressed by logarithmic growth laws, are allowed to reach a stable state of minimal entropy production and, thus, sufficient adaption. Furthermore, our results allow for a simple interpretation of the second law of learning (Eq. (4)). We were able to show that this postulate can be reconciled in the larger context of the principle of minimum entropy production for stable evolutionary systems. Accordingly, this relation describes the slow attainment of evolutionary adaptation to given environmental conditions.

In summary, we have provided a thermodynamic analysis of evolutionary processes. Our results reveal the great similarity between non-equilibrium thermodynamic processes and molecular evolution. However, it should be noted that we have focused solely on species with limited lifespans. Processes such as the birth and death of individuals and their effects on molecular evolutionary processes are therefore not the subject of our discussions. Accordingly, we have interpreted the current genetic information as a pure product of inheritance from the previous generation. The consideration of such events, as well as the consideration of sexual reproduction mechanisms cannot be addressed by our simple approach. However, since these mechanisms are also more complex, it can be assumed that mutational and horizontal gene transfer mechanisms dominated early life on Earth as most simple solutions for molecular evolution. Our results show that the goal of simple evolutionary processes is to achieve a reasonable number of adaptable variables or genetic information in terms of a stable evolutionary equilibrium. In addition, sufficient genetic adaptation can be viewed as a stable evolutionary state that exhibits the highest level of adaptation to given environmental conditions. We hope that our simple approach stimulates further research in this field, as molecular evolution is one of the most fascinating problems in the biological world.

## Data Availability

Not applicable.

## Appendix A: Continuum Equations

In general, continuum equations provide insights into spontaneous changes and conservation or balance relations, respectively, for the variables of interest. In agreement with conservation laws (De Groot and Mazur 1984) for the internal energy, we define the continuum equation for the additive average fitness in terms of

A1 $$\begin{aligned} \rho \frac{d \hat{u}}{dt} + {\nabla }_{\textbf{L}} {\textbf{Q}} = 0 \end{aligned}$$

with the fitness flux ${\textbf{Q}}$, the number of individuals density $\rho = N/\Omega$ and the differential operator ${{\nabla }}_ L = (\partial /\partial {\textbf{L}}_j)$. Here and in the following, vectors are marked by bold symbols and letters. Notably, all further contributions such as external forces are ignored in Eq. (A1). In more detail, Eq. (A1) states that the additive fitness can change due to fluxes in and out of the system. As can be seen, there is no source term in the considered subsystem $\Omega$ that causes a change in the additive fitness. Accordingly, it can be assumed that the fitness properties can only be carried in and out of the system, which in this context already has a connection with gene transfer from the subsystem to its environment and *vice versa*. In addition, the continuum equation for the number of adaptable variables reads

A2 $$\begin{aligned} \rho \frac{d\hat{k}}{dt} + {\nabla }_{\textbf{L}} \textbf{J} = \breve{k} \end{aligned}$$

with the flux of adaptable variables $\textbf{J}$ and the source term $\breve{k}$. The source term accounts for all internal changes in the number of adaptable variables. Accordingly, one can also assume that genetic information is brought in and out of the system but can also spontaneously occur in the subsystem. Such an assumption is in good agreement with genetic mutation effects which may randomly change the number of adaptable variables and, thus, the genetic information..

## Appendix B: Limiting Cases

As already mentioned in the main text, we will evaluate Eq. (15) for several limiting expressions. As a first approach, we focus on the restricted presence of one single individual in the subsystem with volume $\Omega$.

### B.1: One Single Individual

Accordingly, Eq. (19) reduces to

B3 $$\begin{aligned} \sigma = \omega \rho \frac{\mu _{\tilde{N}}}{T} \frac{S}{b} \end{aligned}$$

for $N=1$ as limiting expression due to the assumption ${\nabla }_{\textbf{L}}\mu _{\tilde{N}} = 0$. Here, it is assumed that the single individual is isolated in subsystem $\Omega$ such that any gradient in the evolutionary potential vanishes. This also means that the evolutionary potential of the individual stays constant. However, the actual entropy production is a consequence of the previous population growth rate and the actual learning entropy *S* which expresses the amount of information in terms of the actual number of adaptable variables. As was shown in Vanchurin et al. 2022a, the evolutionary potential can be written as

B4 $$\begin{aligned} \mu = \frac{dU}{dK} \end{aligned}$$

with the empirical relation (Vanchurin et al. 2022a)

B5 $$\begin{aligned} U = a S^n e^{\frac{b}{S}K} \end{aligned}$$

including the free factors *n* and *a*. These factors are closely related to loss functions which can be used to monitor the progress of evolution (Vanchurin et al. 2022a). However, we do not need to discuss these parameters in more detail, as they are not affecting the discussions in the remainder of this article. More details on this discussion can be found in Vanchurin et al. 2022a. Differentiation of *U* with the number of adaptable variables *K* yields

B6 $$\begin{aligned} \mu = \frac{dU}{dK} = ab\, S^{n-1}e^{\frac{b}{S}K} = ab\, S^{n-1} N^E \end{aligned}$$

after consideration of Eq. (2), which can be inserted in Eq. (B3) according to

B7 $$\begin{aligned} \sigma = - \frac{\omega \rho a\, S^{n}}{T} e^{\frac{b}{S}K}. \end{aligned}$$

As can be seen, all parameters have positive values, such that $\sigma \le 0$. This clearly shows, that the entropy production is negative and remains constant over the course of time. However, there are two ways to change the entropy production in the system. One possibility is cell division, as is known for bacteria, which can lead to the formation of further individuals in the system under consideration, thus, making the sum rule of Eq. (19) valid again. The other possibility is spontaneous mutation and, thus, the change in the number of adaptable variables within an individual. Both possibilities may contribute to the entropy production of the system, but for the first case it becomes clear that also the first term in Eq. (19) becomes relevant whenever the number of adaptable variables between the individuals differ.

### B.2: Two Individuals with Small Growth Rates $\omega \rightarrow 0$

As another limiting case in terms of vanishing growth rates $\omega \rightarrow 0$, one can see that Eq. (19) reduces to

B8 $$\begin{aligned} \lim _{\omega \rightarrow 0} \sigma = -\frac{{\textbf{J}}_1}{T} {\nabla }_{\textbf{L}}\left( \mu _1-\mu _{\tilde{N} }\right) \end{aligned}$$

for $N=2$ with species 1 and $\tilde{N}$. This can be related to two individuals of one species in the considered system volume. Moreover, corresponding conclusions as were drawn for the limiting case in Section B.1 apply. With the definition of the evolutionary potential (Eq. (B6)), it follows

B9 $$\begin{aligned} \mu _1-\mu _{\tilde{N}} = ab\, S^{n-1} \left( e^{\frac{b}{S}K_{N}}\left( e^{\frac{b}{S}\Delta K_{1\tilde{N}}}-1\right) \right) \end{aligned}$$

with $K_1 = K_{\tilde{N}} + \Delta K_{1\tilde{N}}$. Insertion into Eq. (B8) yields

B10 $$\begin{aligned} \lim _{\omega \rightarrow \infty } \sigma = -ab\, S^{n-1}\frac{{\textbf{J}}_1}{T} {\nabla }_{\textbf{L}}\left( e^{\frac{b}{S}K_N}\left( e^{\frac{b}{S}\Delta K_{1\tilde{N}}}-1\right) \right) \end{aligned}$$

which can be transformed via ${\nabla }_{\textbf{L}} = (\partial /\partial \Delta K_{i\tilde{N}})(\partial \Delta K_{i\tilde{N}}/\partial {\textbf{L}}) = (\partial /\partial \Delta K_{i\tilde{N}})\cdot {\textbf{u}}_{i\tilde{N}}$ to

B11 $$\begin{aligned} \lim _{\omega \rightarrow \infty } \sigma = -ab^2\, S^{n-2} e^{\frac{b}{S}K_1} \frac{{\textbf{J}}_1}{T}\cdot {\textbf{u}}_{1\tilde{N}} \end{aligned}$$

where the dot product ${\textbf{u}}_{1\tilde{N}}\cdot {\textbf{J}}_1$ is either positive or negative with regard to the value of $\Delta K_{1\tilde{N}}$. Moreover, all other parameters have positive values, such that $\lim _{\omega \rightarrow \infty } \sigma \le 0$. Accordingly, we have introduced a directed flux of adaptable variables or genetic information between the two individuals as expressed by the aforementioned dot product. Accordingly, this expression corresponds to transferred information between individuals which can be loosely associated with horizontal gene transfer. However, it also becomes clear that increasing fluxes for the reference individual $\tilde{N}$ result in decreasing entropy values and, thus, an increase of meaningful information.

## Appendix C: Thermodynamic Forces and Fluxes and Entropy Production Rates

In general, one can define the entropy production density $P = \int \sigma \; d\Omega$ (De Groot and Mazur 1984), such that

C12 $$\begin{aligned} \frac{dP}{dt} = \int \frac{d\sigma }{dt} \;d{\Omega } \le 0 \end{aligned}$$

which shows that the entropy production rate becomes minimal over the course of time or even vanishes for steady states or after approaching equilibrium (De Groot and Mazur 1984). This is a consequence of certain considerations regarding thermodynamic stability and the corresponding values for the entropy production (De Groot and Mazur 1984). Furthermore, one can write the entropy production in accordance with

C13 $$\begin{aligned} \sigma = \sum _{A=1}^C J_A X_A = \sum _{A=1}^{C}\sum _{B=1}^C L_{AB} X_A X_B \end{aligned}$$

with the thermodynamic forces *X*, the thermodynamic fluxes *J* and the Onsager coefficients $L_{AB}$ for *C* non-equilibrium contributions (De Groot and Mazur 1984). In more detail, this description shows that entropy production is driven by fluxes and forces as already discussed in the previous subsections. The forces are usually gradients in the intrinsic variables such as chemical potentials, inverse temperatures or pressure among others. The non-vanishing contributions of the gradients induces fluxes in order to balance the gradients. In consequence, this approach shows that non-equilibrium processes and fluxes usually relax and vanish when the gradients in the intrinsic variables and, thus, the thermodynamic forces decay. This relation between forces and fluxes can also be seen by $J_A = \sum _{B=1}^N = L_{AB}X_B$ which highlights that a thermodynamic force $X_B$ induces a flux $J_A$. In accordance with  (14) for $C=1$, it, thus, follows $\sigma = L_{11}X_1 X_1$, where $X_1$ can already be identified from Eq. (15) as $X_1 = {\nabla }_{\textbf{L}}\left( \mu _i-\mu _{\tilde{N}} \right)$. In addition, one can see from the relation $J = L_{11} X_1$ (De Groot and Mazur 1984), that

C14 $$\begin{aligned} {\textbf{J}}_i = L_{11} {\nabla }_{\textbf{L}}\left( \mu _i-\mu _{\tilde{N}} \right) = L_{11} \frac{b}{S}e^{\frac{b}{S}K_i} {\textbf{u}}_{i\tilde{N}} \end{aligned}$$

in agreement with Eq. (20). Accordingly, the previous relation can be interpreted as an evolutionary flux which is caused by gradients in the evolutionary potential between the reference and the remaining individuals. Further identification of the term on the right hand side reveals that the number of adaptable variables $K_i$ plays a decisive role. With regard to the relations

C15 $$\begin{aligned}{} & {} {\nabla }_{\textbf{L}} = \frac{\partial }{\partial \Delta K_{i\tilde{N}}}\cdot {\textbf{u}}_{i\tilde{N}}, \end{aligned}$$

C16 $$\begin{aligned}{} & {} {\textbf{u}}_{i\tilde{N}} = \frac{\partial \Delta K_{i\tilde{N}}}{\partial {\textbf{L}}} \end{aligned}$$

and the definition

C17 $$\begin{aligned} \Delta \mu _{i\tilde{N}} = \mu _i-\mu _{\tilde{N}}, \end{aligned}$$

the expression for the flux of adaptable variables (Eq. (C14)) can also be written as

C18 $$\begin{aligned} {\textbf{J}}_i = L_{11} \frac{\partial \Delta \mu _{i\tilde{N}}}{\partial \Delta K_{i\tilde{N}}}\cdot {\textbf{u}}_{i\tilde{N}} \end{aligned}$$

which demonstrates that the differences in the evolutionary potentials and the differences in the number of adaptable variables between two species are the main driving factors. In agreement with our previous considerations, this relation clearly shows that evolutionary fluxes are driven by derivatives of gradients in the differences of the evolutionary potentials and the corresponding differences in the number of adaptable variables. In accordance, one can assume that the flux compensates for the gradients in the evolutionary potentials which means a change in the number of adaptable variables.

In general, the entropy production rate is governed by the entropy production coming from the thermodynamic forces and fluxes and can be written as

C19 $$\begin{aligned} \frac{dP}{dt} = \frac{d_XP}{d t} + \frac{d_JP}{d t} \end{aligned}$$

where the subscript *m* in $d_m/d t$ denotes either the differentiation of the flux (*J*) or the thermodynamic force contributions (*X*) in agreement with Eq. (C13). The detailed evaluation in combination with Eq. (C13) yields

C20 $$\begin{aligned} \frac{d_XP}{dt} = \int _{\Omega }L_{11} X_1 \frac{dX_1}{dt} d\Omega = \int _{\Omega } L_{11} \frac{dX_1}{dt} X_1 d\Omega = \frac{d_JP}{dt} \end{aligned}$$

which results in

C21 $$\begin{aligned} \frac{dP}{dt} = 2 \frac{d_XP}{dt} \le 0. \end{aligned}$$

after consideration of Eq. (C20) and Eq. (C12) and in agreement with thermodynamic assumptions (De Groot and Mazur 1984). The previous relation allows us to study the presence of stabilities or pattern formation as well as the temporal behavior of evolutionary systems. Moreover, it shows that the entropy production rate becomes more negative or even vanishes over the course of time.

Finally, one can define the entropy production density (De Groot and Mazur 1984) after application of Eq. (15) in combination with Eq. (16) and Eq. (C12), which results in

C22 $$\begin{aligned} \frac{dP}{dt} = -2 \int _{\Omega }d\Omega \left[ \sum _{i=1}^{N-1} \frac{{\textbf{J}}_i}{T} {\nabla }_{\textbf{L}}\left( \frac{\partial }{\partial t}\left( \mu _i-\mu _{\tilde{N}} \right) \right) + \sum _{i=1}^{N} \frac{\rho S}{bT}\frac{\partial }{\partial t}\left( \mu _i \frac{\partial }{\partial t}\log N^E\right) \right] \end{aligned}$$

giving under the assumption of the exponential growth law (Eq. (17)) the following relation

C23 $$\begin{aligned} \frac{dP}{dt} = -2 \int d\Omega \left[ \sum _{i=1}^{N-1} \frac{{\textbf{J}}_i}{T} {\nabla }_{\textbf{L}}\left( \frac{\partial }{\partial t}\left( \mu _i-\mu _{\tilde{N}} \right) \right) + \sum _{i=1}^{N}\frac{\omega }{T} \frac{\rho S}{b} \frac{\partial \mu _i}{\partial t} \right] \end{aligned}$$

and thus

C24 $$\begin{aligned} \frac{dP}{dt} = -2 \int d\Omega \left[ ab\, S^{n-1} \frac{{\textbf{J}}_1}{T} \cdot {\nabla }_{\textbf{L}}\left( \frac{\partial }{\partial t} (N^E_1-N^E_{\tilde{N}}) \right) + \frac{\omega \rho a\, S^{n}}{T} \frac{\partial }{\partial t} \left( N^E_1+ N^E_{\tilde{N}}\right) \right] \end{aligned}$$

with $N_i^E = \exp (b/S K) = \mu _i/(abS^{n-1})$ for $N=2$ individuals.

## Appendix D: Influence of Logarithmic Growth Laws on the Entropy Production Rates

Insertion of the logarithmic growth law (Eq. (25)) into Eq. (16) yields

D25 $$\begin{aligned} \breve{k} = \frac{\rho S}{b} \frac{ \omega (Z-1)}{e^{\omega t}+Z-1} \end{aligned}$$

which can be inserted into Eq. (14) according to

D26 $$\begin{aligned} \sigma = - \frac{\mu _i}{T} \frac{\rho S}{b} \frac{ \omega (Z-1)}{e^{\omega t}+Z-1} \end{aligned}$$

under the assumption of $N=1$. The further evaluation of the second term of the entropy production rate under consideration of Eq. (B6) yields

D27 $$\begin{aligned} \frac{dP}{dt} \propto - 2\int _{\Omega }d\Omega \left[ \frac{a\rho S^n}{T} \frac{\partial }{\partial t}\left( \frac{ N^E\omega (Z-1)}{e^{\omega t}+Z-1}\right) \right] \le 0. \end{aligned}$$

which gives after insertion of the integrated logarithmic growth law from Eq. (25) in terms of

D28 $$\begin{aligned} N^E = N^E_0\frac{Z e^{\omega t}}{N^E_0(e^{\omega t}-1)+Z} \end{aligned}$$

the following expression

D29 $$\begin{aligned} \frac{dP}{dt} \propto - 2\int _{\Omega }d\Omega \left[ \frac{a \rho S^n}{T} \left( \frac{\omega N_0^E Z e^{\omega t}(Z+1)}{(N_0^E(e^{\omega t}-1)+Z)(e^{\omega t}+Z-1)}\right) \phi (t)\right] \le 0 \end{aligned}$$

with

D30 $$\begin{aligned} \phi (t) = \left( \omega - \frac{\omega e^{\omega t}(N_0^E (e^{\omega t} + Z -1)+(\omega N_0^E(e^{\omega t}-1)+Z))}{(N_0^E(e^{\omega t}-1)+Z)(e^{\omega t}+Z-1)} \right) \end{aligned}$$

such that $dP/dt \propto e^{-\omega t}$ and hence

D31 $$\begin{aligned} \lim _{t\rightarrow \infty }\frac{dP}{dt} = 0 \end{aligned}$$

when combined with the first term of Eq. (19). Thus, limited growth laws lead to vanishing entropy production rates.
