# Correction: Principles of Molecular Evolution: Concepts from Non-equilibrium Thermodynamics for the Multilevel Theory of Learning

**DOI:** 10.1007/s00239-024-10228-2

**Published:** 2025-01-06

**Authors:** Jens Smiatek

**Affiliations:** Institute for Computational Physics, University of Stuttgart, Allmandring 3, 70569 Stuttgart, Germany

**Correction to: Journal of Molecular Evolution (2024) 92:703–719** 10.1007/s00239-024-10195-8

The article "Principles of Molecular Evolution: Concepts from Non-equilibrium Thermodynamics for the Multilevel Theory of Learning," written by Jens Smiatek, was originally published electronically on the publisher’s internet portal on 29th August 2024 without open access. With the author(s)’ decision to opt for Open Choice the copyright of the article changed on 01st December 2024 to ©The Author(s) 2024 and the article is forthwith distributed under a Creative Commons Attribution 4.0 International License, which permits use, sharing, adaptation, distribution and reproduction in any medium or format, as long as you give appropriate credit to the original author(s) and the source, provide a link to the Creative Commons licence, and indicate if changes were made. The images or other third party material in this arti cle are included in the article’s Creative Commons licence, unless indicated otherwise in a credit line to the material. If material is not included in the article’s Creative Commons licence and your intended use is not permitted by statutory regulation or exceeds the permitted use, you will need to obtain permission directly from the copyright holder. To view a copy of this licence, visit http://creativecommons.org/licenses/by/4.0. Open access funding enabled and organized by Projekt DEAL.

